# Sustained Release Technology and Its Application in Environmental Remediation: A Review

**DOI:** 10.3390/ijerph16122153

**Published:** 2019-06-18

**Authors:** Lili Wang, Xiaowei Liu

**Affiliations:** 1 Environmental Engineering, Jiyang College of Zhejiang A & F University, Zhuji 311800, China; 2 Institute of Water Resources & Ocean Engineering, Ocean College, Zhejiang University, Hangzhou 310058, China; 3 Institute of Municipal Engineering, College of Civil Engineering and Architecture, Zhejiang University, Hangzhou 310058, China

**Keywords:** sustained release technology, classification, preparation methods, environmental remediation

## Abstract

Sustained release technology is a class of technology characterized by slowly-releasing specific active substances into a target medium to keep a certain concentration in the system within valid time. As a new of type technology, it has been extensively applied to medicine, chemical engineering, agriculture, environmental protection, etc. The principles and classification of sustained release technologies, as well as typical preparation methods of sustained release agents, were summarized in this paper; by introducing applied research progress of sustained release technologies into environmental fields like rainwater purification, sewage/drinking water treatment, and soil and atmosphere remediation, application features of these sustained release technologies were evaluated, and their application prospect in environmental remediation, especially in water treatment, was predicted.

## 1. Introduction

Sustained release technology (SRT) is a technology taking specific measures to slow down the release of specific active substances (SASs) within a certain time so that designed concentration of SASs can be kept within the system [1]. This technology can also be called release-control technology; it releases at a constant preset speed within the preset time so that SASs are kept within the effective concentration range. Sustained release technology has been widely applied to medicine, chemical engineering, agriculture, and water treatment, etc. In the medical field, it is mainly used to improve compliance of drug administration, reduce toxic and side effects and the stimulation of gastrointestinal tract, improve bioavailability, and decrease total drug dose; in the chemical field, it operates mainly in the form of controlled-release sustained release agent, usually as a constituent in treating fluids like aqueous rust preventive, anti-rust oil, mildew preventive, and stabilizer [2]; in the agricultural field, it is taken as a slow release agent for pesticides in the microcapsule form so that capsule-core pesticides will not be influenced by external temperature, oxygen, ultraviolet ray, etc. [3,4]. In the field of environmental remediation, toxic and harmful substances in the environmental medium are degraded by taking advantage of the small dose size and high efficiency of the sustained release agents [5,6,7,8].

Sustained release technology can be divided into physical sustained release and chemical sustained release [9,10]. For physical sustained release technology, skeleton materials of sustained release agent will not react with active substances and only act as regulators of active substance delivery. There are three common types of physical sustained release agents: (1) uniform type where active substances are uniformly dispersed in skeleton material sustained release agent; (2) capsule type formed by active substances which are wrapped in the sustained release agent; and (3) gel type where sustained release speed is controlled by regulating gel hydration degree. Gel type has been extensively applied in sustained drug release [11], and capsule type is mostly studied at present is coating type technology. Regarding capsule type, the layer of coating material can avoid active substances’ excessive contact with the external environment so as to improve their stability. Regarding the chemical sustained release, polymer is used as the sustained-release agent, and active substances are connected in pre-formed polymer through chemical bonds to form a new compound. A reactive group is needed between active substances and sustained release agent in a chemical sustained release. This group is formed through the fracture of chemical bonds in the system. As a general rule, fracture modes of chemical bonds include hydrolysis and biological hydrolysis. The release rate of active substances depends on the bonding structure, properties, and their degradability [12].

This review aims to provide a reference for the actual application of sustained release technology to environmental remediation. Our paper is organized as follows: We first review the principles and classification of SRT. Subsequently, preparation methods of sustained release agents are discussed in detail. The progress of the application of SRT in practical environmental remediation is then explored. Finally, recommendations to promote SRT applications in environmental remediation are provided.

## 2. Classification of Sustained Release Agents

In the actual application of SRTs, active components and sustained-release materials are usually assembled into sustained release agents with certain physical shapes. Common sustained release agents include tablets, capsules, film agents, microspheres, pellets, etc. [13].

### 2.1. Tablets

Tablets refer to circular or irregular flaky solid agents prepared by blending active components with proper ingredients [14,15]. They have been extensively utilized in sustained release and controlled release agents because of the simple preparation procedure. For instance, drugs—isosorbide mononitrate sustained-release tablets and nifedipine release controlled tablets belong to this category [16]. With the continuous development of macromolecular technologies, coating tablets have dominated a certain status in a sustained-release agent. Coating tablets mainly depend on the coating for protecting tablets from being influenced by air, oxygen, and moisture so that they can maintain long-term stability, and the sustained release goal of active components in the target medium can be reached [17].

### 2.2. Capsules

Capsules refer to solid agent prepared by packing active components in hollow hard capsules or sealing them in elastic soft capsules [18,19,20]. They have been extensively applied due to their advantages that they can cover bad taste and odor of active components, improve their stability and solidify liquid-state active components [21]. For instance, in the preparation process of Ibuprofen sustained release agent, Zhao et al. [22] suspended particles by pumping compressed air in spray-drying coating machine and coated particles using ethylcellulose acetone solution to prepare Ibuprofen particles. Taking chitosan and sodium alginate as carriers, Jia et al. [23] used a complex coacervation method to prepare Etoposide microcapsule particles and packed them into ordinary capsule shells. Xia et al. [24] blended ingredients like sodium carboxymethylcellulose, stearic acid, and sodium bicarbonate with diclofenac sodium, packed the mixture into empty capsule shells, heated them and then prepared Diclofenac sodium gastric floating-type sustained-release tablets. Yang et al. [25] used the film-coating method to prepare acemetacin controlled-release pellets and adopted this pellet to prepare acemetacin-containing capsules. Martinac et al. [26] used chitosan, poly [(α,β-(*N*-2-bis-hydroxyethyl-dl-asparagine)) and poly [α,β-(*N*-3-hydroxypropyl-dl-asparagine)] to make microsphere skeleton and used gemfibrozil as a model drug to prepare sustained release microspheres. Zhang et al. [27] successfully prepared eugenol-gelatin-chitosan nanocapsules employed gelatin and chitosan as carriers by the self-assembly method.

### 2.3. Microcapsules

Microcapsules are formed by coating solid or liquid active components (capsule core materials) with natural or synthetic macromolecular compound materials (capsule materials) taken as capsule film wall shell) [28,29]. If active components are dissolved and (or) dispersed in macromolecular materials, skeleton-type microsphere entities will be formed, which are called microspheres. The particle size of microcapsule and microsphere are within the range of 1–250 μm, so they belong to the micron order and are jointly referred to as microparticles. For instance, Manjanna et al. [30] took alginate as the hydrophobic carrier and calcium chloride as a cross-linking agent to prepare Aceclofenac sustained release microspheres. According to sustained release performance and favorable biocompatibility of alginate, Mankala et al. [31] adopted alginate as a coating material to prepare mucous film microcapsules and employed sodium carboxymethylcellulose as mucous film adhesive. High micro-encapsulation efficiency and good adhesion were obtained. The two were blended in a proper proportion to realize sustained-release drug administration. Fan et al. [32] took gelatin and Arabic gum as capsule materials to prepare econazole nitrate microspheres. Feng et al. [33] prepared leuprorelin sustained release microspheres by taking degradable polymer polylactic acid as the carrier. Taking gelatin as the capsule material, Yang et al. [34] prepared propafenone in sustained-release microcapsules using simple coacervation method. Taking hydroxyethyl methacrylate-methyl methacrylate as the capsule material, Vallbacka et al. [35] prepared microcapsules which could secrete dopamine. By means of layer-by-layer assembly technique, An et al. [36] used human serum albumin and L-α-dimyristoylphosphatidic acid as capsule shell materials to encapsulate ibuprofen for drug-controlled release. Manna and Patil [37] also utilized layer-by-layer assembly technique to prepare encapsulated enamel which is used sodium dodecyl sulfate and chitosan as raw materials.

### 2.4. Film Agent

Films refer to thin-film agents processed by dissolving or uniformly dispersing drugs into film-forming materials [38,39,40]. Yan and Zou [41] prepared compounds with protective films and drug films and then used polyvinyl alcohol, glycerin, and water as protective film components and Arabic gum powder, tinidazole, sodium bicarbonate, tetracaine, bezoar detoxicating tablet, azone, glycerin, and water as drug film components to prepare tinidazole one-way sustained release films. Zhou [42] prepared tinidazole sustained release films by taking tinidazole, sodium carboxymethylcellulose, and polyvinyl alcohol as film materials.

## 3. Preparation Methods of Sustained Release Agents

Sustained release agents can be divided into skeleton type and reservoir type [13]. Drugs are uniformly dispersed in various carrier materials in forms of molecules or microcrystals and microparticles, thus forming skeleton-type sustained release agents; drugs are wrapped in macromolecular polymer films, thus forming reservoir-type sustained release agents. The formulation methods and principles of typical sustained release agents are summarized in Table 1.

## 4. Application of Sustained-Release Technology in Environmental Remediation

With socio-economic and industrial development, water pollution, soil pollution, and atmospheric pollution problems are becoming increasingly prominent. For the sake of pollution control, the governments of many countries have formulated various policies and scientific researchers are actively carrying out applied research regarding environmental remediation. Studies show that sustained-release technology can be used for the remediation of underground water, sewage, drinking water, rainwater, soil, and atmosphere.

### 4.1. Underground Water Remediation

As for underground water pollution problems, Li et al. [64] conducted a pilot-scale study on remediation of underground aquifer polluted by hydrochloric ether using zero-valent iron-sustained-release carbon technology. By injecting a zero-valent iron-sustained-release carbon agent (7200 kg) into the aquifer at underground 9~18 m and conducting regular monitoring of pollutant concentration, it was found that removal rates of 1,1-dichloroethane, 1,2-dichloroethane, and chloroform in the water reached 87.57%, 99.97%, and 99.07%, respectively, after seven months, and their half-life time periods were 115 days, 46 days, and 70 days, respectively. In-situ sustained release of oxidant has also been extensively applied to underground water remediation [65,66]. Chokejaroenrat et al. [67] investigated the degradation of methyl orange by zero-valent activation of persulfate (PS). PS was dosed by PS sustained-release material. Results indicate that, in the presence of PS sustained-release material (paraffin:PS mass ratio = 1:3) and zero-valent iron (paraffin:zero-valent iron mass ratio = 1:4.7), a large quantity of PS was released at the beginning and the concentration reached 5000 mg/L. Within 50 h, 100 mg/L methyl orange could be effectively removed. Therefore, this sustained-release technology can be taken as a pollutant control method for a long time.

A substantial amount of research indicates that potassium permanganate (KMnO_4_) can be applied to underground water remediation as a green oxidizing agent. To improve the utilization efficiency of KMnO_4_, Zeng et al. [68] used environmentally friendly materials paraffin and silica sand as composite materials to prepare a composite-type KMnO_4_ sustained-release solid through the melting–forming method. The study results indicated that the sustained release quantity of KMnO_4_ gradually increased with time. Cumulative release percentages of KMnO_4_ reached 28.3% and 58.8% respectively at 13 days for sustained-release KMnO_4_ with paraffin:silica = 1:6. This sustained-release material could realize sustained and controlled release of KMnO_4_. Wang et al. [69] used a KMnO_4_ sustained-release agent to degrade landfill leachate and found that the chemical oxygen demand (COD) removal rate could reach as high as 57.1% with the minimum precipitation, which was only 8.5% of the rate when pure KMnO_4_ was added. Besides, existing studies have found that biodegradable macromolecular materials can be used as coating carriers to lengthen the service life of oxidizing agents and improve underground water remediation efficiency [70]. Yang et al. [71] prepared a kind of sustained-release carbon source composite material, using hemp fiber, polybutylene succinate, and polyethylene as raw materials, for the remediation of nitrate-polluted underground water. The nitrate-nitrogen removal rate can be maintained above 96.0% during 66 days of operation.

### 4.2. Sewage Treatment

Urban sewage treatment, an important constituent part of environmental protection, is essential to protect the local ecological balance, improve natural conditions, and eliminate environmental pollution. Some scholars have used new types of solid sustained-release carbon sources to reinforce nitrogen removal by denitrification of sewage and the denitrification rate increased by about 10% [72,73,74]. Besides, Xu et al. [75] developed a novel sustained-release dephosphorization tablet to enhance sewage treatment by anaerobic-oxic (A/O) process. This sustained-release dephosphorization tablet is slowly dissolved under scouring action of water flow and meanwhile, it maintains a certain strength and shape. When its dosage is two tablets (200 g/each tablet) per ton of sewage, 82–87% treatment efficiency could be reached. This study pointed out that removal efficiency was expected to be further elevated if sludge backflow in the system could be effectively improved. Chen [76] proposed a sewage treatment method with ecological concrete. The sewage passes through a plug flow-type ecological concrete water treatment facility after pretreatment. This ecological concrete treatment facility realizes water purification by doping with sustained-release water purification material in the ecological concrete. After treating with this purification device, COD and biochemical oxygen demand (BOD) of a heavily polluted river water decreased by above 50%. The removal rate of total phosphorus (TP) exceeded 70% and that for total nitrogen reached 20%. Cavallaro et al. [77] explored the separation of oil from aqueous solution with alkanoate-modified halloysite nanotubes (HNTs). An adsorption capacity of 2.6 g n-decane per 100 g HNTs at 23 °C was obtained, indicating the potential for such modified HNTs in oil-polluted water treatment. Zhao et al. [78] studied the treatment efficiency of dye-polluted water with HNTs. The model dyes, cationic rhodamine 6G and anionic chrome azurol S, showed good adsorption on HNTs. The maximum adsorption capacities of rhodamine 6G and chrome azurol S were 43.6 mg/g and 38.7 mg/g, respectively. As compared with kaolinite, HNTs showed two times higher adsorbancy.

### 4.3. Drinking Water Treatment

In recent years, raw water pollution and secondary pollution of pipe network water have posed a severe challenge to drinking water safety. To cope with this challenge, sustained-release technologies make their contributions. Sustained-release disinfectant containing silver compounds has been used as a new type of water treatment agent to inhibit virtue growth and corrosion [79,80]. Hu et al. [81] developed a kind of silver-loaded diatomite. When silver content was 1.46%, it could completely kill Escherichia coli in a water sample within 30 min, and leaking amount of silver was always lower than 50 μg/L no matter it was soaked or filtered. As a safe and effective antibacterial material, this silver-loaded diatomite was considered to be promising in drinking water treatment. Wang [82] prepared silver-loaded activated carbon with the vacuum impregnation method. When the silver loading quantity was 0.97 wt%, it could kill Escherichia coli at a concentration of 10^7^ CFU/mL within 120 min. This method can realize the sustained release of silver and thus maintain a high sterilizing effect. Therefore, it could be classified as a new type of advanced drinking water treatment technology.

Additionally, taking a new type of sustained-release disinfection tablet as the study object, Yang et al. [83] evaluated its effect on killing Escherichia coli and controlling the total bacterial count in drinking water. Results indicated that the prepared disinfection tablet could realize the effective control of Escherichia coli and the total colony count when the valid concentration of chlorine is ≥0.3 mg/L at contact time 30 min. Furthermore, changes in water quality parameters like pH, water temperature, and chromaticity had no significant influences on the disinfection effect. Ding et al. [84] also developed a new type of sustained-release solid chlorine dioxide disinfectant for drinking water treatment. Compared to traditional chlorine dioxide disinfectant, it had the advantages of stability, convenient storage and transportation, and long-acting time, etc. Giving the freshwater shortage on coastal cities and frontier defense, which solved drinking water problems mainly by transporting and storing water, Li et al. [85] developed a sustained-release disinfectant, which was prepared by blending stable dichloride isocyanuric acid with macromolecular adhesive in a certain proportion. This sustained-release disinfectant had a long sustained release time, generally 8–13 days. Field experiments proved that it could efficiently disinfect stored water for over consecutive 25 days upon a single administration. With respect to the Cr(III) pollution in drinking water, research scholars have proposed an emergency nondestructive remediation process, which used a sustained-release ferric chloride-magnetic module as a purification agent [86]. Study results show that the mesitite-magnetic module has the best remediation ability of Cr(III). Cr(III) in surface water can be treated to satisfy drinking water standards within 3 h under normal conditions, and the treatment process generates no obvious harm to organisms.

### 4.4. Rainwater Treatment

In the past few years, the concept of a sponge city has been proposed. With the goal of saving water resources and improving the urban ecological environment, many researchers hope to purify rainwater runoffs before they infiltrate into the soil to conserve underground water or are reused to supplement municipal water (such as landscape water) [87,88]. With a reference to the mature experience of America in rainwater conservation and initial rainwater purification, Gao et al. [89] designed a set of Chinese rainwater conservation and utilization and initial rainwater purification systems, which solved initial rainwater purification problems while conserving rainwater. It realized initial rainwater purification mainly through soil conservation and purification, plant purification, natural precipitation, permeation, and filtering. Based on this design concept, many scholars have conducted research on rainwater sustained-release technologies. For instance, a rainwater collection and sustained-release device for the root area of trees has been invented, which can balance water use in the rainy season and dry season, relieve water use pressure in the dry season, and reduce water transport and lower plantation cost [90]. In the meantime, slow underground permeation can greatly improve water utilization efficiency. Lin et al. [91] found in their study that the water absorbent polymer—phenolic aldehyde foamed plastic, when added into environmental mineral material, could improve rainwater purification capacity to a great extent. When 10% bentonite was added, the removal rate of BOD_5_ in rainwater could be elevated to 90% and COD removal rate to 80% with the purification effect of turbidity reaching 83%. Moreover, as water absorbent polymer had a good water-absorbing property, water-retaining property, and sustained-release effect, this material could realize rainwater purification and storage.

### 4.5. Soil Remediation

Sustained-release technology has also found its application in soil remediation. Zhong et al. [92] invented a sustained-release fertilizer with a soil remediation effect, which was prepared by blending biodegradable poly-hydroxyalkanoates with organic and inorganic nutrient substances. Under the action of soil microorganisms, poly-hydroxyalkanoates can be slowly degraded. The degradation of products can provide nutrient substances needed by soil microbial growth and, thus, promote heavy metals ionization by soil microorganisms. Furthermore, these degradation products can also act as nutrient substances for plants to promote plant growth and improve soil remediation efficiency. Liu et al. [93] used a gradient dilution method to screen out mixed bacteria which can degrade. The mixed bacteria were then immobilized onto turfy soil using adsorption method. Results indicated that turfy soil immobilized with mixed bacteria can function as a microbial sustained-release agent. Application of this microbial sustained-release agent made the degradation rate of petroleum hydrocarbon in polluted soil (30 g/kg oil content) increase from 24.3% (the case of degrading bacteria free) to 28.4% for a 30-day remediation time.

In view of alpine and arid climate and zinc-deficient soil structure in plateau areas of China, researchers have found that palygorskite, a natural ecological material, features adsorptivity, a sustained-release property, dispersity, suspension property, and displacement property, so it can be used for fertilizer release control, soil property improvement, polluted soil remediation, and saline land improvement, etc. [94]. Xue et al. [95] proposed using palygorskite and papermaking waste derivatives (lignosulfonic acid) as raw base materials to prepare multicomponent and multifunctional sustained-release zinc fertilizer through the solution polymerization method. With functions that include releasing zinc fertilizer, absorbing water, preserving soil moisture, and improving soil, this product can be applied to technical fields of eco-environmental protection and agricultural fertilizers. This technology can contribute to realizing waste recycling and reducing manufacturing and provide a reference for the application of sustained-release micro-fertilizers in the ecological rehabilitation engineering field.

In recent years, phytoremediation technology has been considered to be an important means of remediating polluted soil and is continuously applied to practice. Researchers found that sustained-release complexing agent can increase collective absorption of heavy metals by plants, thus, significantly improving phytoremediation efficiency [96,97].

### 4.6. Indoor Air Purification and Atmospheric Pollution Remediation

As for atmospheric pollution, biological methods have been confirmed to be efficient [98,99,100,101]. For example, biological filtration is an effective method of removing harmful and repugnant substances in the exhaust gas. Wang et al. [102] developed a biological filler of exhaust gas with sustained-release function. This filler is characterized by high nutrient content, good air permeability, and strong load and impact resistance. Nutrient substances of the filler can slowly release through the synergistic effect of dissolution and diffusion and microbial degradation. This biological filter can be stably used for a long time. Zhang et al. [103] developed a sustained-release agent that takes paraffin and rosin as the sustained-release matrix and calcium carbide as the biomethane inhibitor and investigated diffusion laws of the effective inhibitory component-acetylene. Results showed that when the mass fraction of rosin in the matrix was 20% and the mass ratio of matrix and calcium carbide was 1:1, the hardness and compactness of the sustained-release matrix were improved along with a diffusion coefficient of acetylene reached 2.3 × 10^−8^ cm^2^·min^−1^ (R^2^ = 0.9901). Experimental results proved that this sustained-release agent could effectively reduce the emission of biomethane from artificial sources like a municipal solid waste landfill.

For indoor air pollution problems, research scholars have developed chlorine dioxide sustained-release air purification device. When chlorine dioxide release rate is 7.4 mg·h^−1^ and relative humidity is in the range of 29–31%, 59–61%, and 89–91%, it can effectively purify hazardous substances like formaldehyde generated from interior decoration [104]. Although slowly released chlorine dioxide gas can effectively eliminate odor indoor and disinfect the air, chlorine dioxide itself is toxic. Therefore, this technology is being continuously improved so that it can be applied with as few side effects as possible [105].

## 5. Conclusions

Sustained-release technology has achieved certain progress in the aspect of environmental remediation, but the present sustained-release technologies are mainly physical or/and chemical processes-triggered, environmentally sensitive, and not real-time controllable. The future sustained-release technologies should develop towards the accurate, quantitative, biotechnology-integrated, and intelligent sustained-release direction with efforts put into solving biodegradability and recycling problems of carrier materials. As to the water treatment field attracting extensive attention at present, some technical barriers that exist in the implementation process, such as secondary pollution and interface passivation, must be addressed. Therefore, the high-performance, economical, non-toxic, harmless, second-pollution-free, photo-triggered, and smart sustained-release agents will have a broad application prospect in the environmental remediation field.

## Figures and Tables

**Table 1:** Formulation methods and principles of typical sustained release agents.

Morphology of Sustained Release Agent	Preparation Method	Preparation Principles and Features
**(Micro)capsule**	Complex coacervation	Two macromolecular molecules with opposite charges are taken as composite capsule materials which cross-link with each other under certain conditions and experience coacervation with capsule core to form capsules [43].
	Simple coacervation	Coagulant is added in macromolecular capsule material solution to reduce macromolecular solubility for coacervation into capsules [44].
	Solvent/non-solvent method	According to the solubility principle, target substance is firstly dissolved in a solvent, followed by necessary operations, and then nonsolvent chemicals are added so that target substance is precipitated out in the form of crystallization or wrapping on other material surfaces [45].
	Drying in liquid technique	The volatile solvent in the disperse phase is removed from the emulsion to prepare microcapsules (microspheres) [46].
	Spray drying method	After thinner is atomized in a drying room, moisture will be rapidly vaporized when thinner contacts hot air so as to obtain the dry product. This method can directly dry solution and emulsion into powdery or particulate products, thus saving evaporation, crushing and other procedures.
	Spray congealing	After being dissolved with a proper solvent, the drug is blended with the molten carrier, and the product can be obtained after cooling and congealing.
	Self-assembly method	The principle of molecular self-assembly is to use molecular recognition between a molecule pair or fragment pair to form a class of molecular polymers with a specific structure, stability, and specific properties through non-covalent interaction [47].
	Fluidized bed coating	Capsule core is suspended in the coating room through vertical strong airflow. Capsule solution is sprayed onto the surface of capsule core through a nozzle so that hot airflow resulting in the suspension of capsule core volatilizes the solvent until it becomes dry, and then thin capsule film is formed on the surface to obtain microspheres.
	Multiorifice-centrifugal process	Drug obtain centrifugal force through a high-speed revolution cylinder. The drug solution passes through the capsule material at a high speed to form a liquid film which is then solidified through different methods to obtain microspheres [48,49].
	Supercritical fluids	The drug solution is dispersed in supercritical fluid through the nozzle of a supercritical fluid device, the organic solvent is dissolved in a supercritical fluid and then extracted, and the remaining drug is formed into microspheres [50,51].
	Spinning disk atomization	The material enters a disk spinning at a high speed through the material supply tube. Under high-speed shear action, it leaves the edge of the disk and solidified after being cooled at the bottom, thus forming microspheres [52].
	Interface polycondensation	Capsule film is generated due to monomer polycondensation reaction on the interface of the disperse phase (aqueous phase) and continuous phase (organic phase), thus forming microspheres [53].
	Chemical radiation	γ-ray energy generated by ^60^Co is used for cross-linking and congealing of polymer (gelatin or polyvinyl alcohol) so as to form microspheres [54].
**Adsorption of sustained release solid**	Adsorption method	Active substances are adsorbed through many pores or high specific surface area to reach the sustained release effect [55,56].
**Tablets/particles**	Drying or wetting method for particle/tablet preparation	Skeleton material, drug, and other ingredients are blended to directly prepare particles/tablets, and in some cases, the adhesive or wetting agent is used to help the particle/tablet preparation [57,58].
**Solid dispersion**	Solid dispersion method	The drug is incorporated into a solid carrier under a highly dispersed state through certain methods (melting method, solvent method, and mechanical dispersion method) [59].
**Nanoparticles**	Emulsion polymerization	The monomer is firstly dispersed in aqueous capsule emulsion droplets containing an emulsifier, it is polymerized into nanoparticles using methods such as high-energy radiation and then turned into the solid state after phase separation, and then solid nanoparticles are prepared [60,61].
	Natural polymer condensation method	Natural macromolecular (like protein and polysaccharides) solution containing active components is added into the oil phase and then water/oil emulsion is formed through mechanical agitation or ultrasonic dispersion. Macromolecules are condensed through chemical crosslinking, thermotropy or dehydration by salting-out under proper conditions, thus forming nanoparticles [62,63].
